# Botulinum Toxin Injection into the Digastric Muscle: Current Clinical Use and a Report of Five Cases

**DOI:** 10.3390/biomedicines11102767

**Published:** 2023-10-12

**Authors:** Alina Ban, Raluca Roman, Simion Bran, Mihaela Băciuț, Cristian Dinu, Emil Crasnean, Oana Almășan, Mihaela Hedeșiu

**Affiliations:** 1 Department of Maxillofacial Surgery and Radiology, Iuliu Hațieganu University of Medicine and Pharmacy, 37 Iuliu Hossu Street, 400029 Cluj-Napoca, Romania; 2 Department of Oral and Maxillofacial Surgery and Implantology, Iuliu Hațieganu University of Medicine and Pharmacy, 37 Iuliu Hossu Street, 400029 Cluj-Napoca, Romania; 3 Department of Prosthetic Dentistry and Dental Materials, Iuliu Hațieganu University of Medicine and Pharmacy, 32 Clinicilor Street, 400006 Cluj-Napoca, Romania

**Keywords:** botulinum toxin, digastric muscle, orthognathic surgery, malocclusion, angle Class II

## Abstract

The present research aimed to review the clinical applications of botulinum toxin-A (BTX-A) injection into the anterior belly of the digastric muscle (ABDM) and to highlight the potential role of the BTX-A injection into ABDM in preventing postsurgical relapse. Five Class II malocclusion patients who underwent orthognathic surgery received BTX-A injections into both ABDM for the prevention of postoperative relapse. The relapse was evaluated using lateral cephalometric radiographs by comparing the postoperative cephalometric analyses at two different time points, postoperatively at 2 weeks (T_1_), and long-term, at 9 months after the surgical intervention (T_2_). The results demonstrated no significant differences between T_2_ and T_1_ for the Selle-Nasion-point A (SNA) angle, Selle-Nasion-point B (SNB) angle, point A-Nasion-point B (ANB) angle, mandibular length, and sagittal mandibular position. The patients exhibited stable occlusion without any signs of relapse after the surgery. A single BTX-A injection into the ABDM can effectively prevent postoperative relapse in Class II malocclusion patients, following orthognathic surgery. From a clinical perspective, in case of optimal dosage and procedure, BTX-A injection could be considered as the primary option for the prevention of postsurgical relapse for Class II malocclusion patients.

## 1. Introduction

The digastric muscle is a suprahyoid muscle (SH) with two separate muscular bellies, an anterior one and a posterior one, which are connected by an intermediate tendon. The anterior belly (ABDM) is attached to the digastric fossa and runs toward the hyoid bone. The posterior belly (PBDM) attaches to the temporal bone and the mastoid process and then reaches the intermediate tendon. The tendon penetrates the stylohyoid muscle and also passes through the fibrous loop, which is attached to the body and greater cornu of the hyoid bone [1]. Its contraction opens the mouth, pulls the mandible downward, and elevates the hyoid bone during mastication and swallowing [2].

Botulinum toxin-A (BTX-A) is a metalloprotease produced by Clostridium Botulinum and a powerful therapeutic tool for clinical maxillofacial applications [3]. Its action consists in inhibiting acetylcholine release at neuromuscular junctions, being a safe and effective approach to reduce muscular activity by relaxing the striated muscles [4].

In the maxillofacial area, BTX-A has been used as a cosmetic agent, especially for the removal of wrinkles and the reduction of muscular volume [5]. In recent years, indications of BTX-A injections have increased, being also reported in the treatment of myofascial pain [6], temporomandibular disorders (TMDs) [7], temporomandibular joint (TMJ) dislocation [8], bruxism [9], prevention of open bite [10], or treatment of plate fractures [11].

The effect of BTX-A injections lasts from three to six months. New neuromuscular junctions and axonal connections are established, which gradually replace the non-functional ones. Muscle strength is recovered between three to six months [12].

Orthognathic surgery could induce dimensional changes of the ABDM, associated with increased length and decreased width, as muscular alterations are often implicated in surgical relapse [13]. To avoid recurrence corrections for Class II malocclusion, different surgical techniques have been discussed. The majority of the reviewed studies focused on muscular alteration by myotomies of the suprahyoid muscles [14,15]. However, this technique is invasive and can generate postoperative complications, such as swelling and bleeding [16].

As BTX-A injections induce muscular weakness, some authors showed that BTX-A injection into the ABDM can correct an open bite caused by a bilateral mandibular angle fracture [10]. A single dose of BTX-A injection into the ABDM can be a useful method in preventing surgical relapse in patients with deep-bite and open-bite malocclusion, treated with orthognathic surgery [17,18].

The present research aimed to review the clinical applications of BTX-A injection into ABDM and to highlight the potential role of the BTX-A injection into ABDM in preventing postsurgical relapse in Class II malocclusion patients.

## 2. Materials and Methods

The planning and preparation of this study have followed the guidelines established by the PRISMA declaration [19] for the preparation of systematic reviews and meta-analyses.

### 2.1. Eligibility Criteria

The inclusion criteria were as follows: BTX-A injections into the digastric muscles, patients older than 18 years, and articles published in the English language.

The exclusion criteria were articles outside the area of interest of this study, in vitro studies, studies where BTX-A injection was performed into other mandibular muscles than the digastric, animal studies, and papers with no full-text access.

### 2.2. Search Strategy

An electronic search in three medical databases, including Pubmed, Scopus, and Embase, was performed on the 5 May 2023, with no restriction on the publication period. MeSH terms were used where applicable. The search included the following keywords: “botulinum toxin” and “digastric muscle”. The full search strategy is presented in Table 1.

### 2.3. Study Selection

The study selection was performed using Rayyan online software [20], allowing the organization of the publications and to perform an independent, blind screening of the included studies. Two researchers (A.B. and R.R.) then evaluated the titles and abstracts for relevance, followed by a full-text review of the retrieved articles. A subsequent manual search of all the selected studies, carried out by reading the references included in the list, was also performed. In case of a disagreement between authors, a third researcher (E.C.) mediated discussions and provided consultation.

### 2.4. Statistical Analysis of the Case Series

The clinical records of five adult patients (3 females, 2 males) who underwent orthognathic surgery for Class II malocclusion, between June 2019 and September 2021, in the Department of Maxillo-Facial Surgery of Cluj-Napoca, were considered in this case series. The mean age of all patients was 28.4 ± 13.2 years. All patients received bimaxillary surgery, namely, Le Fort I osteotomy and bilateral sagittal split osteotomy (BSSO), with an average amount of mandibular advancement of 9.3 mm.

The statistical data analysis was conducted using IBM SPSS Statistics 26.0 software for Windows (SPSS Inc., Chicago, IL, USA). The normality of the data was tested using the Shapiro–Wilk test. The mean and standard deviation were calculated for the preoperative (T_0_), early (T_1_), and late postoperative (T_2_) cephalometric angles and distances of each patient. The differences between time points were evaluated by paired sample *t*-test. The level of significance was set at *p* < 0.05.

## 3. Results

### 3.1. Data Collection

Fifty-four articles were initially identified from the electronic databases. After excluding duplicate and irrelevant articles, forty-seven full-text articles were considered eligible. For our initial analysis, forty-seven studies were selected based on title and abstract. The screening process generated seventeen articles which were retrieved in full text. Then, one article was not retrieved and another one was eliminated due to the language of publication, resulting in fifteen publications. Later, articles that considered digastric muscle transfer as a treatment option, followed by research showing an unclear injection protocol, as well as technical notes, were excluded. Finally, a total of nine publications were included in this review.

**Figure 1:**
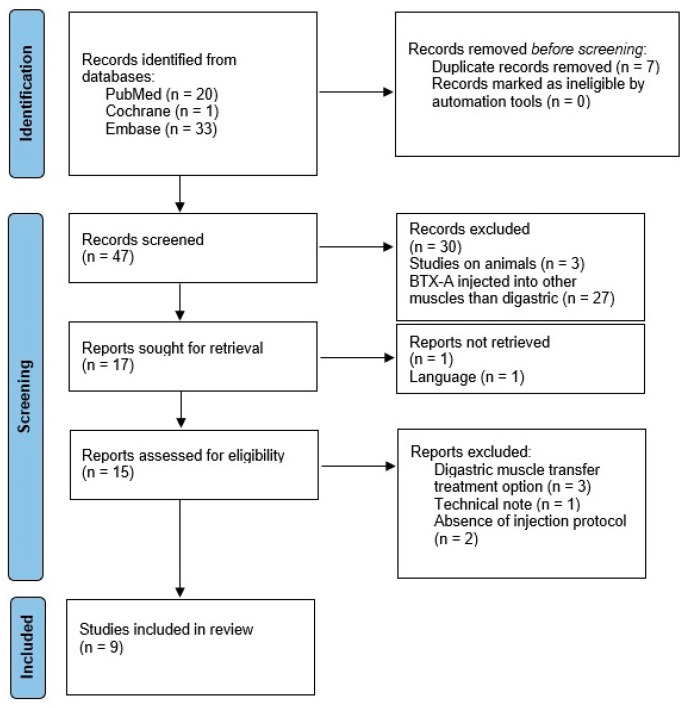
Flowchart of the selected articles and reasons for the exclusion of articles after full-text evaluation.

A flowchart indicating the selected articles and the reasons for the exclusion of articles after full-text evaluation are presented in Figure 1 [19].

### 3.2. Description of the Studies

The extracted data from the studies included: (1) author and year of publication; (2) type of publication; (3) number of enrolled study subjects; (4) diagnosis; (5) area of injection; (6) method; (7) spots of infiltration; (8) number of units injected; and (9) number of sessions involved.

The main characteristics of the studies considered in this review are summarized in Table 2.

### 3.3. Demographic Information

The review comprised 87 patients, resulting from the selected studies. The prospective studies included patients with ages ranging between 28 and 85 years, while the case reports included three patients of 21 years old and one patient of 67. None of the reviewed studies included control groups. Our research comprised patients with ages ranging between 18 and 33 years old.

### 3.4. Protocol of BTX-A Injection

This research was approved by the Institutional Review Board of the University of Medicine and Pharmacy, Cluj-Napoca, Romania, and the study was given approval number 125/19.05.2017.

The clinical records of five adult patients (3 females, 2 males) who underwent orthognathic surgery for Class II malocclusion, between June 2019 and September 2021, in the Department of Maxillo-Facial Surgery of Cluj-Napoca, were considered in this case series. The mean age of all patients was 28.4 ± 13.2 years. All patients received bimaxillary surgery, namely, Le Fort I osteotomy and bilateral sagittal split osteotomy (BSSO), with an average amount of mandibular advancement of 9.3 mm.

Preoperatively, patients were screened via ultrasound (US) for ABDM anatomical variations, to avoid possible complications of BTX-A injection.

Each subject received a total of 20 units of abobotulinum toxin-A (Dysport^®^ Ipsen Limited, Berkshire, UK) into both ABDM, intraoperatively. The injections were performed by the same maxillofacial surgeon, by using a 25 g needle, with five units of BTX-A being injected into two sites of each muscle, through an extra oral approach.

**Figure 2:**
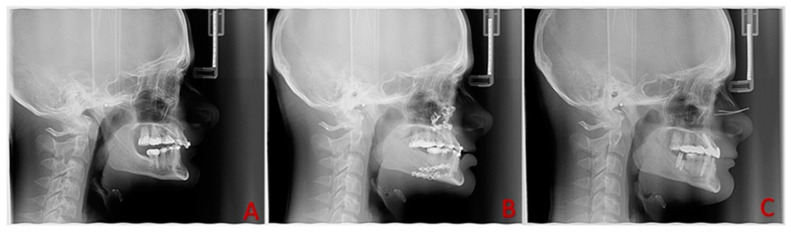
Lateral cephalometric radiographs, (**A**) preoperatively (T_0_), (**B**) at 2 weeks postoperatively (T_1_), and (**C**) at 9 months after the orthognathic surgery (T_2_). BTX-A injection into the ABDM seemed to prevent postoperative relapse. The SNA angle, SNB angle, ANB angle, mandibular length, and sagittal mandibular position were stable postoperatively. Courtesy of Dr. B.S.’s collection.

The protocol also included patient assessment via lateral cephalometric radiography, using the same equipment (Vatech PaX-I SCTM, Hwaseong, Korea), preoperatively (T_0_), postoperatively at 2 weeks (T_1_), and long-term at 9 months after the surgical intervention (T_2_) (Figure 2). Subjects were positioned in the cephalostat, and then the head holder was adjusted until the ear rods could be positioned into the ears without moving the patient. All radiographs were taken in a standardized and reproducible position, the natural head position (NHP), with teeth together and lips in a relaxed condition.

The cephalometric analyses were achieved digitally using NemoCeph v.2021 software. The measurements were performed by a single, blinded, duly calibrated examiner, using the criteria of Roth-Jarabak. The following angles (°) and distances (mm) were assessed: SNA (°), SNB (°), ANB (°), sagittal mandibular position (°), mandibular length (mm), mandibular plane (°), Saddle angle (°), gonial angle (°), articular angle (°), sum int angles (°), Y axis to SN (°), posterior face height (mm), anterior face height (mm), and Jarabak ratio (%) (Table 3).

Table 4 summarizes the mean and SD of the achieved cephalometric measurements and the differences between the time points exemplified by *p*-values.

Preoperative (T_0_) and postoperative (T_1_) SNA angle, SNB angle, ANB angle, mandibular length, sagittal mandibular position, posterior face height, and Jarabak ratio were statistically significant.

The results demonstrated no significant differences between T_2_ and T_1_ for the SNA angle, SNB angle, ANB angle, mandibular length, and the sagittal mandibular position.

The patients exhibited stable occlusion without any signs of relapse after surgery and were appreciative of the treatment’s aesthetic outcomes.

## 4. Discussion

Nine relevant studies were included in this review; among these, four were prospective studies, four were case reports, and one was a retrospective case note review.

Clinical applications of BTX-A

In three of the included studies [10,18,27], patients received BTX-A injection into the ABDM muscle as an adjunctive therapy for orthognathic and trauma surgery, in order to prevent postoperative relapse. It is known that skeletal stability following mandibular advancement is a controversial topic in the literature, with various factors being implicated in postoperative relapse [28]. An early relapse could be explained by an improper postoperative bony interference on sagittal split ramus osteotomy techniques, an improper condylar position, or an excessive torque of the condyle [11]. Nevertheless, among them, the increased tension of the paramandibular soft tissues, including SH, could be a decisive factor in the relapse [29]. These findings are strengthened by a study published in 2021 [13], which showed that ABDM suffers postoperative dimensional changes, in terms of thinning and elongation, with these findings being also incriminated in the surgical relapse.

The postoperative relapse could also be explained by the connected muscles’ primary influence on the position of the mandible. Jaw-closing muscles are mainly attached to the ramus, while jaw-opening muscles are attached to the body [30]. In the case of BSSO, the mandibular body is disconnected from the ramus. However, fixation of the segments restores the anatomy of the mandible, and the jaw-opening muscles could track the mandible posteroinferiorly [31]. In the present study, five patients with Class II malocclusion who underwent bimaxillary orthognathic surgery were included. The surgical technique involved maxillary Le Fort I osteotomy and BSSO, together with BTX-A injection into ABDM, intraoperatively. The protocol of our study followed the same concept demonstrated by van der Linden et al. [30], attempting to cancel possible traction of the mandible by injecting BTX-A into ABDM.

Nonetheless, the intention of our study was not to increase the stability of osteosynthesis but to decrease the ABDM activity by using BTX-A. This approach is analogous to previous studies that assessed the role of myotomies in preventing postoperative relapse in patients with Class II malocclusion [14,15]. However, the value of suprahyoid myotomies has not been proven [29]. The explanation behind our study protocol stems from the fact that postoperatively, the stretching of the ABDM could track the mandible posteroinferiorly. This gives rise to a constant force opposite to the vector of the mandibular advancement, which might be the reason for the sagittal repositioning of the mandible over time [31].

The results of our research showed significant differences between T_1_ and T_0_, in the case of the SNA angle, SNB angle, ANB angle, mandibular length, sagittal mandibular position, posterior face height, and Jarabak ratio, demonstrating that maxillary and mandibular movements with bimaxillary osteotomy were effective on hard tissues in both vertical and horizontal directions. Still, the sagittal relapse was evaluated by comparing the SNA angle, SNB angle, ANB angle, mandibular length, and sagittal mandibular position at T_2_, with the same landmarks at T_1_. We found no significant differences between T_2_ and T_1_ in the case of SNA angle, SNB angle, ANB angle, mandibular length, and sagittal mandibular position, indicating a stable occlusion without any signs of postoperative relapse.

Another factor that affects postoperative relapse is the magnitude of horizontal mandibular advancement, and it is reported that advancements of 7 mm and more are more susceptible to horizontal recurrence [32]. Supporting this finding, our case series included patients with an average amount of mandibular advancement of 9.3 mm. Therefore, we sought to emphasize the therapeutic usefulness of the BTX-A injection into the ABDM in cases of orthognathic patients with severe Class II malocclusions and mandibular advancement of more than 7 mm. The other studies included in this systematic review targeted different pathologies. The study protocols were different, but all revolved around the same final aim, the chemodenervation of the digastric muscle, in order to obtain positive results in the case of anterocollis (AC) [21], facial synkinesis [22], or Meige syndrome [23,24]. Research published in 2021 used BTX-A as a treatment for AC posture, a complex subtype of cervical dystonia and the most infrequent of its abnormal head positions [21]. The authors understood the importance of the role of the deep cervical floor (DCF), SH, and sternocleidomastoid muscles (SCM) in all head and neck flexion movements. To our knowledge, this is the first research that implies injecting the three groups of flexor muscles (DCF, SCM, SH) at the same time, in order to control this dystonic movement. Pescarini et al. [22] showed the benefits of BTX-A injection into the PBDM, in postparalytic facial synkinesis with persistent tightness and aching around the angle of the jaw, in patients who do not respond to maximal treatment aimed at the platysma. This was the first study to demonstrate the potential role of PBD chemodenervation in managing patients with postparalytic synkinesis. Additionally, another piece of research demonstrated a positive response to an unusual focal position-sensitive alternating tremor of the jaws to BTX-A injection into the digastric and masseter muscle [25].

Two other recent studies highlighted a status improvement in patients with Meige syndrome to BTX-A injection into the superficial SH [23,24]. Meige syndrome is a rare pathology that consists of idiopathic blepharospasm and oromandibular dystonia [33]. Speech or breathing disturbances were also reported. To date, there is no curative treatment for Meige’s syndrome, with the BTX-A injections being considered the most effective remedy [34].

Another application of the BTX-A injection concerned the treatment of myogenic compression of the internal jugular vein, as discussed by Klocheva et al. [26]. The authors tried to diminish the clinical manifestations of this condition by injecting BTX-A into the PBDM.

Injection methods

The injection method of BTX-A involved muscle palpation, the guidance of electromyography (EMG), US, or both. Studies that included the injection of several deep muscle groups, such as the digastric, considered that EMG and US are both safe and effective methods for injecting BTX-A [21,23,24,25,26]. A technical note published in 2015 emphasized the importance of screening for ABDM anatomical variations before BTX-A injection in order to prevent undesired adverse effects of BTX-A injection [35]. Screening for ABDM could be achieved via US, CT, and MRI [36,37]. Our research conducted a preoperative US screening of the ABDM, and then a palpatory, intraoperative identification of the ABDM, using the digastric fossa, which was in concordance with the findings of Zdilla et al. [35].

The majority of the studies included in this review performed a bilateral injection of the digastric muscles (ABDM or PBDM), except for two studies in which facial synkinesis and internal jugular vein compression were treated [22,26]. This could be explained by the fact that the pathologies involved in a bilateral approach are characterized by a bilateral contraction of specific muscular groups of the SH, a bilateral chemodenervation being, therefore, required.

Dosage variability

The dosage of the BTX-A varied largely, although this is regarded as an extremely important factor affecting complications. BTX-A doses are generally adjusted equivalent to factors such as age, number of muscles involved, severity of the muscle’s hyperactivity, and previous response to BTX-A injection into the digastric muscle. The suggested doses of toxin ranged from 5 to 70 units, depending on the method presented. In our case series, we used 20 units of BTX-A. The BTX-A was injected bilaterally, into the ABDM, through a metal syringe needle, and aspiration was performed at the time of injection in order to avoid intravenous injection. If BTX-A is injected into the blood vessel, there could be no effect on the muscle, possibly causing severe complications such as dysphagia and respiratory distress [38].

To date, there are no clear protocols regarding the BTX-A injection into the digastric muscles. The optimal dosage should be demonstrated using follow-up research. However, our case series showed that BTX-A injection into the ABDM is relatively safe compared to suprahyoid myotomy, a procedure with high morbidity [14,15].

Nowadays, different types of BTX-A products are used to treat a range of conditions, including cervical dystonia, hemifacial spasm, bruxism, masseter hypertrophy, or TMDs. The three main BTX-A products currently marketed worldwide are onabotulinumtoxin A, abobotulinumtoxin A, and incobotulinumtoxin A [39]. There are important pharmacological differences between these preparations, related to both their manufacture and formulation, which may affect clinical activity [40]. Our review did not find a significant association between the use of a particular BTX-A product and a specific diagnosis. Although both the dosage and measurement units are different, it must be considered that switching from an approved effective dose of one BTX-A product to another product raises the issue of dose ratio and plays an important role in understanding that different types of products can have varying degrees of effectiveness and can provide different clinical outcomes.

Originality of the study

The originality of our study lies in the fact that it presents targeted BTX-A chemodenervation of the ABDM to prevent postoperative relapse in patients with Class II malocclusion who need orthognathic surgery. To our knowledge, this is the first research to highlight the potential role of ABDM chemodenervation in managing postoperative relapse in Class II malocclusion patients. Postoperative skeletal stability represents a key element of any surgical correction. Surgical advancement of the mandible causes stretching of the paramandibular soft tissues, including the digastric muscle [29]. This may create a continuous force opposite to the vector of the mandibular advancement, which is an important factor for the postoperative sagittal readjustment of the mandible [6].

Based on these findings, BTX-A injection into the ABDM was used for skeletal stability, in the case of Class II malocclusion patients, following orthognathic surgery. The present study has limitations. First of all, our research included only five patients and the effect of BTX-A injection was difficult to assess. Therefore, a large-scale case–control study would be required for definite conclusions. Secondly, the optimal dosage is not fully acknowledged, being a critical factor affecting complications.

## 5. Conclusions

A single BTX-A injection into the ABDM shows promising results in preventing postoperative relapse in Class II malocclusion patients, following bimaxillary orthognathic surgery. To date, there are scarce data, with only three papers aiming at the use of BTX-A injection into the ABDM in orthognathic surgery, and therefore an optimal dosage and a clear protocol should yet be established by using further larger data research.

## Figures and Tables

**Table 1:** Search strategy for each database.

PubMed (n = 20)
“botulinum toxins”[MeSH Terms] OR (“botulinum”[All Fields] AND “toxins”[All Fields]) OR “botulinum toxins”[All Fields] OR (“botulinum”[All Fields] AND “toxin”[All Fields]) OR “botulinum toxin”[All Fields] “digastric”[All Fields] OR “digastrics”[All Fields] “muscle’s”[All Fields] OR “muscles”[MeSH Terms] OR “muscles”[All Fields] OR “muscle”[All Fields]
Cochrane (n = 1)
“botulinum toxins”[MeSH Terms] OR (“botulinum”[All Fields] AND “toxins”[All Fields]) OR “botulinum toxins”[All Fields] OR (“botulinum”[All Fields] AND “toxin”[All Fields]) OR “botulinum toxin”[All Fields] “digastric”[All Fields] OR “digastrics”[All Fields] “muscle’s”[All Fields] OR “muscles”[MeSH Terms] OR “muscles”[All Fields] OR “muscle”[All Fields]
Embase (n = 33)
(‘botulinum toxin’/exp OR ‘botulinum toxin’ OR (botulinum AND (‘toxin’/exp OR toxin))) AND digastric AND ‘digastric muscle’

**Table 2:** The main characteristics of the studies included in the review.

Authors, Year of Publication	Type of the Study	Patients (n)	Part of Digastric	Dose (U)	BTX-A	Diagnosis	Method	Spots of Infiltration	Number of Sessions	Conclusion
Seok et al., 2013 [10]	case report	1	ABDM	20	Meditoxin Type A	posttraumatic anterior open bite	palpation	2 spots/bilaterally	1	“BTX-A injection into the anterior belly of the digastric muscle successfully corrected post-traumatic open bite”
Coclici et al., 2021 [13]	prospective	5	ABDM	20	Abobotulinumtoxin A	Class II malocclusion	palpation	2 spots/bilaterally	1	“the postoperative muscular changes indicated consistency and potential benefit of using BTX-A in reducing the risk of surgical relapse”
Kang et al., 2019 [18]	case report	1	ABDM	20	Meditoxin Type A	Class II malocclusion/open bite	NA	2 spots/bilaterally	1	“BTX-A injection into the anterior belly of the digastric muscle demonstrated postoperative stability in case of class II open-bite patient”
Marion et al., 2021 [21]	prospective	15	ABDM	10–30	Abobotulinumtoxin A	anterocollis	ultrasound and EMG guidance	NA/bilaterally	1	“the key to successfully injecting patients with anterocollis is a joint Neuro-ENT clinic, by focusing on every dystonic muscle, in the same session”
Pescarini et al., 2021 [22]	prospective	33	PBDM	5	Incobotulinumtoxin A	facial synkinesis	ultrasound-guided	1 spot/ipsilaterally	1	“lower lip asymmetry is treated by using chemodenervation or surgery, both methods being clinically efficient”
Fernández-Pajarín et al., 2020 [23]	case report	1	ABDM	15	Incobotulinumtoxin A	Meige syndrome	NA	NA/bilaterally	2	“BTX injections into the hyoid muscles showed good results”
Watson et al., 2021 [24]	retrospective case note review	13	ABDM	10–30	Abobotulinumtoxin A	Meige syndrome	NA	NA/NA	1	“upper airway obstruction from palatal, suprahyoid muscles or tongue base dystonia could cause breathing dystonia”
Tarsy et al., 2006 [25]	case report	1	NA	20	Onabotulinum toxin A	jaw tremor	EMG guidance	1 spot/bilaterally	3	“a patient with jaw tremor was responsive to BTX-A treatment”
Klocheva et al., 2014 [26]	prospective	17	PBDM	50–70	NA	internal jugular vein compression	NA	NA/ipsilaterally	1	“BTX injection could be the optimal treatment for patients with internal jugular vein compression”

**Table 3:** The cephalometric angles (°) and distances (mm) of the included patients.

Measurement	Definition
SNA (°)	the angle between Sella–Nasion–point A
SNB (°)	the angle between Sella–Nasion–point B
ANB (°)	the angle between point A–Nasion–point B
Mandibular length (mm)	the linear distance from Gonion to Menton
Sagittal mandibular position (°)	the angle between Sella–Nasion–Pogonion
Mandibular plane (°)	the angle between the anatomic Frankfurt horizontal plane and the line drawn along Gonion and Menton
Saddle angle (°)	the angle between the anterior and posterior cranial base
Gonial angle (°)	the angle between ramus height and mandibular plane (Ar-Go-Me)
Articular angle (°)	the angle between the posterior cranial base and ramus height (S-Ar-Go)
Sum int angles (°)	sum of angles (Saddle angle + Articular angle + Gonial angle)
Y-axis to SN (°)	the angle connecting Gnathion–Sella–Nasion
Posterior face height (mm)	the linear distance from Sella to Gonion
Anterior face height (mm)	the linear distance from Nasion to Menton
Jarabak ratio (%)	the ratio of the Posterior and Anterior facial height

Sella (S)—the center of the hypophyseal fossa; Nasion (N)—the junction of the nasal and frontal bones at the most posterior point on the curvature of the bridge of the nose; A-point (A)—an arbitrary measure point on the innermost curvature from the maxillary anterior nasal spine to the crest of the maxillary alveolar process; B-point (B)—an arbitrary measure point on the anterior bony curvature of the mandible; Pogonion (Pg)—the most anterior point on the contour of the chin; Menton (Me)—the lowest point on the symphysis of the mandible; Gonion (Go)—a point midway between the points representing the middle of the curvature at the left and right angles of the mandible; condylion (Co); Articulare (Ar)—a point midway between the two posterior borders of the left and right mandibular rami at the intersection with the basilar portion of the occipital bone.

**Table 4:** The mean and SD of the achieved cephalometric measurements and the differences between the time points.

Patient Sample (n = 5)								
Variables	T_0_		T_1_		T_2_		*p*-Value	
	Mean	SD	Mean	SD	Mean	SD	T_1_–T_0_	T_2_–T_1_
Sagittal relation								
SNA (°)	80.8	2.2	81.6	2	81.2	2	0.0161 *	0.1778
SNB (°)	74.6	3.6	78.6	4.8	78.8	4.7	0.00187 *	0.3739
ANB (°)	6	3.7	2.8	4.6	2.6	4.6	0.00284 *	0.3739
Mandibular length (mm)	65.6	2.6	73.4	2	73.2	1.9	0.00082 *	0.3739
Sagittal mandibular position (°)	77.6	4.9	80.4	5.5	80.6	5.3	0.0071 *	0.37
Vertical relation								
Mandibular plane (°)	25.4	14.2	28.6	13	28.4	12.7	1.933191	0.186950
Saddle angle (°)	128	6.7	128.8	8	128.8	7.7	0.202512	0.500000
Gonial angle (°)	116.4	11.3	119.2	6	119.8	7.8	0.207962	0.345153
Articular angle (°)	140.8	14.7	140.6	16.5	139.8	14.2	0.470484	0.324131
Sum int angles (°)	385.4	14.2	388.6	13	388.4	12.7	0.062677	0.081804
Y-axis to SN (°)	66.8	6	65.8	5.9	65.6	5.9	0.115100	0.310654
Posterior face height (mm)	60.3	34.9	58.7	34.1	59.5	34.9	0.029057 *	0.115100
Anterior face height (mm)	81.2	45.3	83.8	46.7	84.4	47	0.115100	0.186950
Jarabak ratio (%)	60.32	35.6	56.5	33.5	56.7	33.6	0.015010 *	0.088904

* Statistically significant comparison of means between early postoperative and preoperative cephalometric measurements (T_1_–T_0_) (*p* < 0.05).

## Data Availability

The data presented in this study are available on request from the corresponding author.
